# Surface charge-dependent cytokine production using near-infrared emitting silicon quantum dots

**DOI:** 10.1038/s41598-024-60536-2

**Published:** 2024-04-26

**Authors:** Shanmugavel Chinnathambi, Naoto Shirahata, Pooria Lesani, Vaijayanthi Thangavel, Ganesh N. Pandian

**Affiliations:** 1 Institute for Integrated Cell-Material Sciences, Institute for Advanced Study, Kyoto University, Kyoto, 616-8510 Japan; 2 Research Center for Materials Nanoarchitectonics, National Institute for Materials Science, 1-1 Namiki, Tsukuba, 305-0044 Japan; 3 Graduate School of Chemical Sciences and Engineering, Hokkaido University, Kita 13, Nishi 8, Kita-Ku, Sapporo, 060-0814 Japan; 4 School of Science, STEM College, RMIT University, Melbourne, VIC 3000 Australia; 5 Koch Institute for Integrative Cancer Research, Massachusetts Institute of Technology, Cambridge, MA 02139 USA

**Keywords:** CpG oligodeoxynucleotides, Immune-stimulation, Silicon quantum dots, Surface charge, Pluronic F-127, Interleukins, Drug delivery

## Abstract

Toll-like receptor 9 (TLR-9) is a protein that helps our immune system identify specific DNA types. Upon detection, CpG oligodeoxynucleotides signal the immune system to generate cytokines, essential proteins that contribute to the body’s defence against infectious diseases. Native phosphodiester type B CpG ODNs induce only Interleukin-6 with no effect on interferon-α. We prepared silicon quantum dots containing different surface charges, such as positive, negative, and neutral, using amine, acrylate-modified Plouronic F-127, and Plouronic F-127. Then, class B CpG ODNs are loaded on the surface of the prepared SiQDs. The uptake of ODNs varies based on the surface charge; positively charged SiQDs demonstrate higher adsorption compared to SiQDs with negative and neutral surface charges. The level of cytokine production in peripheral blood mononuclear cells was found to be associated with the surface charge of SiQDs prior to the binding of the CpG ODNs. Significantly higher levels of IL-6 and IFN-α induction were observed compared to neutral and negatively charged SiQDs loaded with CpG ODNs. This observation strongly supports the notion that the surface charge of SiQDs effectively regulates cytokine induction.

## Introduction

The frequent presence of CpG dinucleotides in the genomes of viruses and bacteria means that synthetic oligodeoxynucleotides (ODNs) comprising them can activate the immune system through their interaction with Toll like receptor-9 (TLR-9)^1^. TLR-9 is a membrane protein characterized by extracellular leucine-rich repeat motifs and a cytoplasmic Toll/interleukin-1 receptor (TIR) signaling domain. It is localized to the endoplasmic reticulum of B cells and antigen-presenting cells^2^. Targeting TLRs has been recognized as a crucial approach to addressing inflammatory diseases, cancer immunotherapy, and subunit vaccines, as these receptors play a significant role in regulating the human innate immune system^3^. CpG ODNs and TLR-9 interact within acidified endosomes, leading to the production of cytokines such as IL-6 and IFN-α. This process occurs when the endosome becomes acidified. Cytokines are proteins that play essential roles in immunity, cellular proliferation, differentiation, apoptosis, and inflammation. Cytokines are substances produced by different cells in the human body, depending on physiological events, that play an essential role in communication between immune cells and other cells. Cytokines are classified into various types, such as growth factors, interleukins, interferons, tumor necrosis factors, and chemokines^4^.

Synthetic CpG ODNs, utilized in clinical applications, are classified into four categories according to their sequence and cytokines-inducing potential. Among these, class A and Class B CpG ODNs have been extensively studied, and their mechanisms and effects are well understood^5^. Activation of IFN-α through TLR-9 occurs mainly in plasmacytoid dendritic cells upon stimulation by Class A CpG ODNs^6^. The Class B CpG ODNs undoubtedly trigger IL-6 induction from B cells while failing to induce IFN-α^7^. Class A CpG ODNs induce IFN-α due to their palindromic and polyguanine sequences that lead to the formation of a higher-order structure. This is in contrast with Class B CpG ODNs, which lack this ability^8^. It's interesting to observe that class B CpG ODNs show the potential to induce IFN-α and IL-6 when bound to nanoparticle surfaces through electrostatic forces. The multimerization of CpG ODNs on nanoparticle surfaces is the reason behind the cytokine induction^9^. This is a promising finding that could lead to further research and development in the field of drug delivery via nanoparticles^10–15^. Various methods have been developed for delivering CpG ODNs using nanoparticles to enhance immunotherapy’s effectiveness. Cationic starch nanoparticles were utilized to enhance antitumor immunity through the delivery of CpG ODNs^16^. Researchers utilized yeast β-glucan-grafted glycogen nanoparticles to achieve precise and effective delivery of CpG ODNs to macrophages for the purpose of immunotherapy^17^. Later, a new delivery system was developed by encapsulating CpG ODNs within zeolitic imidazolate framework-8 (ZIF-8) nanoparticles, taking advantage of its porous structure for high loading capacity^18^. The study conducted by Lai et al. revealed that a promising treatment for hepatocellular carcinoma involves using lipid nanoparticles to deliver IL-12 mRNA therapy. Specifically, this therapy has been found to suppress MYC-driven hepatocellular carcinoma effectively. This discovery presents a potential breakthrough in the field of cancer treatment, as it demonstrates a promising approach to combating one of the most common types of liver cancer^19^. Nanoparticles containing an ionizable lipid have been shown to deliver replicon RNA into cancer cells effectively. This process facilitates gene expression, activates innate immunity pathways, and immunogenic cell death, marking a significant advancement in targeted cancer therapy^20^.

Since their discovery in 1995, unmethylated CpG ODNs have been widely used in basic research, clinical applications, and as therapeutic agents in cancer immunotherapy, viral infection, allergic diseases, and asthma, attributed to their potent immunostimulatory activity. The key factors for clinical translation using CpG motifs are protecting CpG ODNs from DNase degradation and ensuring their targeted delivery to human B-cells and plasmacytoid dendritic cells, which express TLR-9. Consequently, developing efficient, targeted delivery systems for CpG ODNs has emerged as a critical priority. Achieving this not only enhances the therapeutic efficacy of these agents but also paves the way for advanced research and development^21^.

Quantum dots are used for tissue imaging and drug delivery due to their near-infrared emission, which allows for efficient penetration of human tissue^22–24^. In our study, we found that the surface charge of SiQDs is crucial for cytokine induction, which is dependent on the electrostatic linkage of class B CpG ODNs onto the SiQDs. In the past, researchers have utilized quantum dots based on heavy metals for drug delivery and bioimaging^25^. Cd-based QDs are commonly used for bio-imaging due to their high quantum efficiencies. QDs have high brightness and stability of photoluminescence (PL), making them suitable for medical applications such as fluorescence image-guided surgery for tumor removal. However, CdSe-based QDs contain toxic cadmium^26^. The toxicity of different QDs is evaluated through in vivo models, revealing that QDs containing Cd and Lead (Pb) exhibit high toxicity, as expected. Despite displaying good performance in Near Infrared (NIR) PL due to quantum confinement, Ag_2_Se QD also exhibits high cytotoxicity. While these QDs offer benefits in PL performance, the potential risk associated with the accumulation of their constituent elements in the human body outweighs their advantages. We conducted research to produce SiQDs that are approximately 3.6 ± 0.7 nm in size. These SiQDs have the unique ability to emit light in the near-infrared region, which makes them safe for fluorescence labeling and tracking in living organisms without causing any toxicity^27^. These QDs of this specific size undeniably possess properties that are highly valuable for bio-imaging purposes^28^. The surface of non-dispersible synthesized SiQDs was effectively coated with Pluronic-F127, resulting in their complete dispersion. The surface charge of SiQDs was altered using Pluronic-F127 (neutral), amine-modified Pluronic-F127 (positive), and acrylate Pluronic-F127 (negative). Class B CpG ODNs were attached to the surface of these SiQDs, depending on the surface charges. Our findings demonstrate that SiQDs possessing positive charges elicit the production of more IL-6 and IFN-α due to the electrostatic attraction with ODNs as compared to those with negative and neutral charges.

## Materials and methods

### Materials

Triethoxysilane, 4-nitrophenyl chloroformate (NPC), ethylenediamine, and acryloyl chloride were obtained from TCI Japan. 1-Decene, Pluronic F127, methylene chloride, and anhydrous triethylamine from Sigma-Aldrich (St. Louis, MO, USA). Petroleum ether from Wako Pure Chemical Industries, Tokyo, Japan. No modifications or alterations were made to the chemicals before their use, ensuring reliable and accurate results. To obtain pure and deionized water, a Sartorius arium 611 UV water purification system was employed (Sartorius AG, Goettingen, Germany).

### Characterization

The crystalline lattice structure of Decane terminated SiQDs (SiQDs-De) was captured using a high-resolution transmission electron microscopy (HR-TEM) with 300 kV, specifically the Tecnai G2 F30. To observe the samples, they were deposited onto an ultrathin copper grid. The Pluronic coating on SiQDs was measured using an FTIR spectrophotometer. (Thermo Scientific, USA). The spectrophotometer (PerkinElmer Lambda 35, USA) was used to record their absorbance within the range of 200–600 nm. The fluorescence excitation and emission spectra of SiQDs were captured using the JASCO FP-8300 Fluorometer. We measured the emission spectra using different excitation wavelengths with a fixed scan rate of 1000 nm/min. The samples were prepared in a volume of 1.0 mL using Milli-Q water. The absolute photoluminescence quantum yield of surface-modified SiQDs was measured using a Quantaurus-QY spectrometer from Hamamatsu Photonics. The confocal laser scanning fluorescence microscope from Leica Microsystems, Germany, was used for cell imaging. The measurement of cell viability was carried out using a microplate reader to detect the absorption at 450 nm (MTP-880Lab; Corona, Hitachinaka, Japan). The concentration of CpG ODN is measured using a NanoDrop spectrophotometer. (Thermo Scientific, USA). To quantify the surface charge of SiQDs, we utilized a zeta-potential analyzer (LEZA-600, Otsuka, Japan) in water at a concentration of 1.0% w/v.

### SiQDs synthesis

Triethoxysilane (TES) (16 mL) was added to a two-necked round-bottom flask and stirred in an argon (Ar) atmosphere while being cooled in an ice bath. While stirring vigorously, 32 mL of acidic water (pH 3, using 12 M HCl) dropped under an Ar gas flow. The sample was subjected to xerosol formation process, followed by drying it under vacuum to remove any remaining liquid. While stirring with argon flow, acidic water was added slowly to TES. The Xerosol that resulted was formed within two hours. It was then left to dry overnight under a vacuum. The amorphous hydrogen silsesquioxane powder was heated in a furnace at 1150 °C for 3 h (10^-4^ Pa) in a 5%/95% H_2_/Ar flow after being transferred to a quartz crucible. The obtained powder is a solid of uniform dark-brown color. The SiO_2_/SiQD powder samples were produced by mechanically grinding the powder in a mortar.

### Decane-terminated SiQDs (SiQDs-De) synthesis

The SiOx/SiQD powder (300 mg) was mixed with 48% HF (10 mL) and ethanol (10 mL) and slurried in a small Teflon container. The SiOx matrix is removed through an 80-min vigorous stirring hydrofluoric acid etching^29^. The SiQDs, after being hydrogen-terminated, were separated through centrifugation (14,000 rpm, 10 min). Afterward, they were washed twice with ethanol and then isolated once again through centrifugation. The substance was moved to a flask that already had 1-decene inside it. The sample was purged with Ar for 15 min at room temperature and then heated in an Ar atmosphere at 170 °C for 2 hours^30^. The temperature was set up around 170 °C, because of the boiling point of the 1-decene. The mixture was allowed to cool down until it reached room temperature. The SiQDs were coated with decyl and purified using high-performance liquid chromatography with chloroform as the eluent. After the purification process, the SiQD-De material was subjected to vacuum drying to remove any residual liquid. Following the drying process, the SiQD-De was suspended again in toluene (5 mL) to prepare it for further use in subsequent experiments. The quantum yield of the SiQDs-De is 45% and it is measured by a Hamamatsu quantum yield measurement system.

### Synthesis of positive and negative surface charged pluronic F127

To synthesize a product, we dried 20 g of Pluronic F127 in a vacuum oven for six hours. We then diluted the dried Pluronic in 100 mL of methylene chloride and added 240 mg of NPC dropwise to the solution in the presence of anhydrous triethylamine (1.2 mmol). The mixture was then allowed to react for 12 h. After the overnight reaction, we purified the product by precipitating it in an excess amount of petroleum ether. The obtained material was then vigorously dried under a vacuum to procure the desired pure product.

Next, we dissolved 20 g of NPC-Pluronic F127 in 100 mL of anhydrous methylene chloride and added 45 mg of ethylenediamine to synthesize amine end-capped Pluronic F127. We precipitated the resulting product in petroleum ether and dried it under a vacuum.

Lastly, we made an acylated Pluronic copolymer using the following procedure: we diluted 10 g of dried Pluronic F127 and 250 µL of triethylamine in 50 mL of methylene chloride. We then added 125 µL of acryloyl chloride to the solution and mixed it for 24 h at 4˚C. After the reaction, we purified the acrylate Pluronic F127 by precipitating it in an excess amount of petroleum ether, followed by drying it under a vacuum^31^.

#### Pluronic-F127 coated SiQD-De

To prepare the solution, dissolve 200 mg of Pluronic F127 / am-Pluronic F127 / ac-Pluronic F127 in 9 mL of water and add 1 mL of 0.1 M NaCl solution. The solution was agitated for a maximum of two hours to ensure that it was completely dispersed. A vial of Pluronic F127 solution was gently mixed with a 5 mL toluene containing 1 mg of SiQDs-De. The vial was forcefully shaken for several minutes until a well-formed emulsion was created. The solution has been securely placed within a fume hood, facilitating its evaporation. The experiment was completed successfully, as the toluene layer evaporated completely within 48 h. After sonication for a few minutes, the water layer was dialyzed against water using 14 kDa for approximately 2 h to ensure the complete removal of any residual Pluronic F127 and HCl. The final solution was probe sonicated to achieve good dispersion. It appeared milky and required further dilution in water for biomedical applications.

### CpG ODNs loading on pluronic-modified SiQDs

The 72-base (5′ TCGTCGTTTTGTCGTTTTGTCGTTTCGTCGTTTTGTCGTTTTGTCGTTT CGTCGTTTTGTCGTTTTGTCGTT-3′ (72 bases) natural phosphodiester CpG ODN2006 × 3-PD (hereafter CpG ODNs) was diluted to a concentration of 100 µM using sterilized water. The solution was mixed assertively by adding 6 µL of it to 40 mL of 1 mg/mL PSiQDs solution. The mixture was then shaken aggressively for a duration of 4 h. To collect PSiQDs with bound CpG ODNs, the mixture was centrifuged at 15,000 rpm for 15 min. The concentration of unattached CpG ODNs in the supernatant was measured using a spectrometer to determine the amount of CpG ODNs bound onto pluronic-modified SiQDs.

### Cell culture, cytokine, and cytotoxicity assay

We obtained the human peripheral blood mononuclear cells (PBMCs) from Cellular Technology (OH, USA) and the 293XL-hTLR9 cells from Invitrogen (CA, USA). The PBMCs were cultured following the manufacturer's guidelines. The 293XL-hTLR9 cells were cultured in DMEM supplemented with 10% FBS, penicillin, streptomycin, and blasticidin. The cells were placed in a culture maintained at 37 °C with 5% CO_2_.

We performed a cell cytotoxicity kit 8 (CCK-8) assay to evaluate the safety of PSiQDs for cells. To evaluate the impact of PSiQDs on cell viability, cell cultures were carried out using a 96-well plate. Each well was seeded with 5000 cells and exposed to 1 mg/mL of PSiQDs. After incubation for 24 and 48 h, a cytotoxicity assay was performed to determine the impact of PSiQDs on cellular activity.

PBMCs were seeded in a 96-well plate and stimulated with CpG ODNs in the presence or absence of SiQDs. After 48 h, the culture medium was gathered to be used for a cytokine assay. The quantities of IL-6 and IFN-α were measured using enzyme-linked immunosorbent assays (ELISAs). Ready-Set-Go! (EBioscience, USA) and a human IFN-Module Set ELISA (eBioscience, Austria) was used to measure IL-6 and IFN-α levels, respectively.

### Fluorescence microscopy

To determine where CpG ODNs are located inside cells, they were marked with a substance called FITC at the 3’ end. The PSiQDs with CpG ODNs were added to 293XL-hTLR9 cells at a concentration of 80 µg/mL with FITC labeling. After 24 h, the cells were washed three times with PBS and fixed using 3.7% formaldehyde. The lysosome/endosome is stained with an anti-Lysosomal-associated membrane protein 1 (LAMP-1) antibody. The stained PBMC cell images were captured using a confocal microscope.

### Statistics

The cytokine level difference was assessed with a student t-test, and a post hoc Bonferroni correction was conducted for multiple comparisons. If the *P*-value of a result was less than 0.05, then it was considered to be significant.

## Results and discussion

### PSiQDs for CpG ODNs delivery

**Figure 1:**
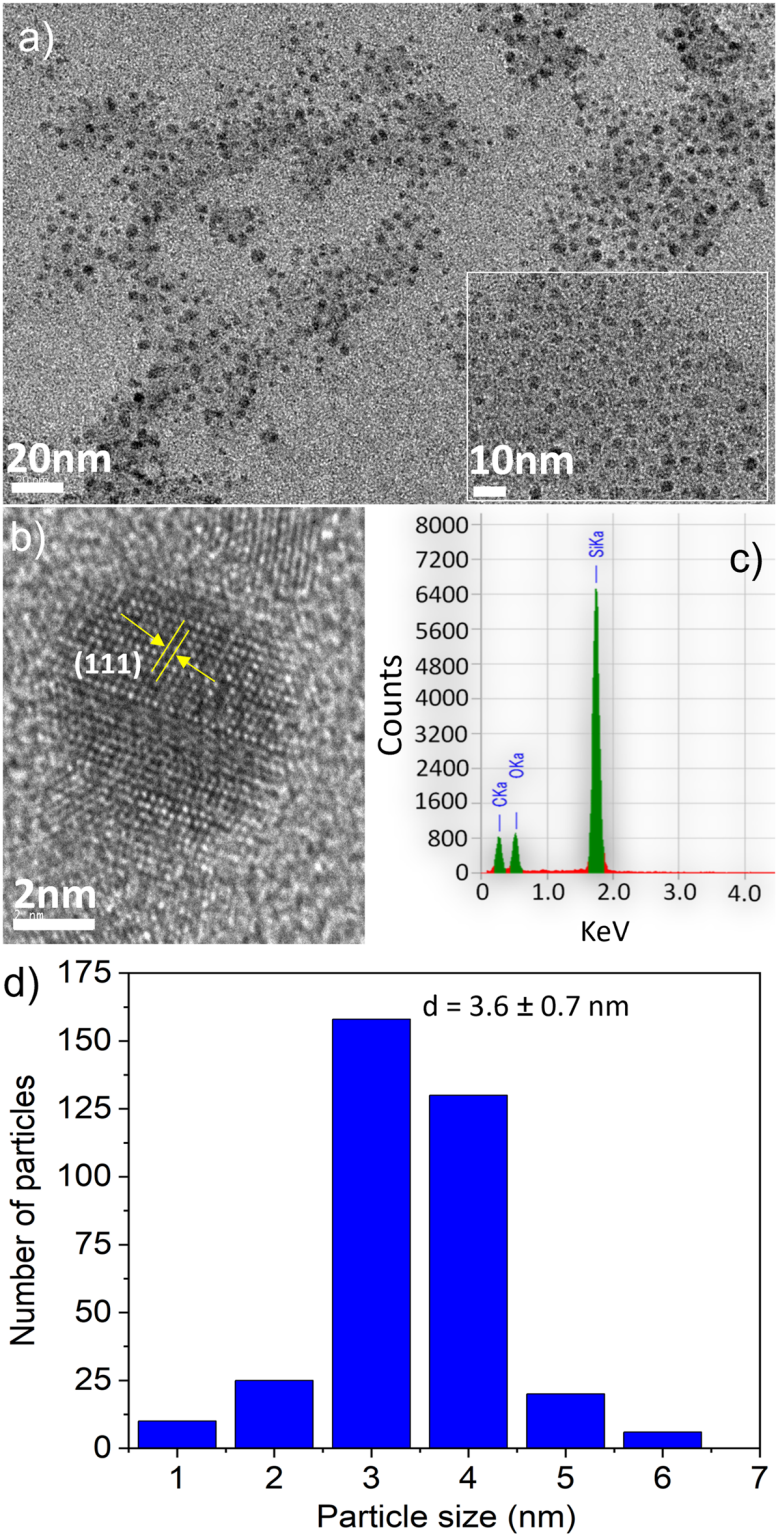
TEM images of SiQDs with two different scales (a), HR-TEM shows the lattice fringe distance match with SiQDs (b), EDS spectra of SiQDs (c), and the average particle size (d).

The SiQDs' native oxide was removed via hydrofluoric acid etching after synthesizing them in low-pressure plasma of Ar and SiH_4_^32^. In order to disperse the SiQDs in water, we modified their surface with Pluronic F127^33,34^, a nonionic surfactant polyol. This surface modification was a crucial step to ensure that the SiQDs-F127 became water-soluble following the synthesis process. The HR-TEM images provided atomic lattice fringes (hkl = 111), confirming the presence of a diamond-like structure (Fig. 1a,b)^35^. Figure S1 displays all the surface-modified TEM images with a 20 nm scale bar, and all the particle sizes fall in between 2–6 nm scale. The micro-Raman spectrum of SiQDs: H (Fig. S2) clearly exhibits a redshifted peak at 520 cm^−1^ in comparison to bulk crystalline silicon (520 cm^-1^). The EDX spectrum revealed the presence of Si, O, and C elements, suggesting that F127 was present on the Si nanoparticles (Fig. 1c). The size of these QDs was approximately 3.6 ± 0.7 nm (Fig. 1d).

**Figure 2:**
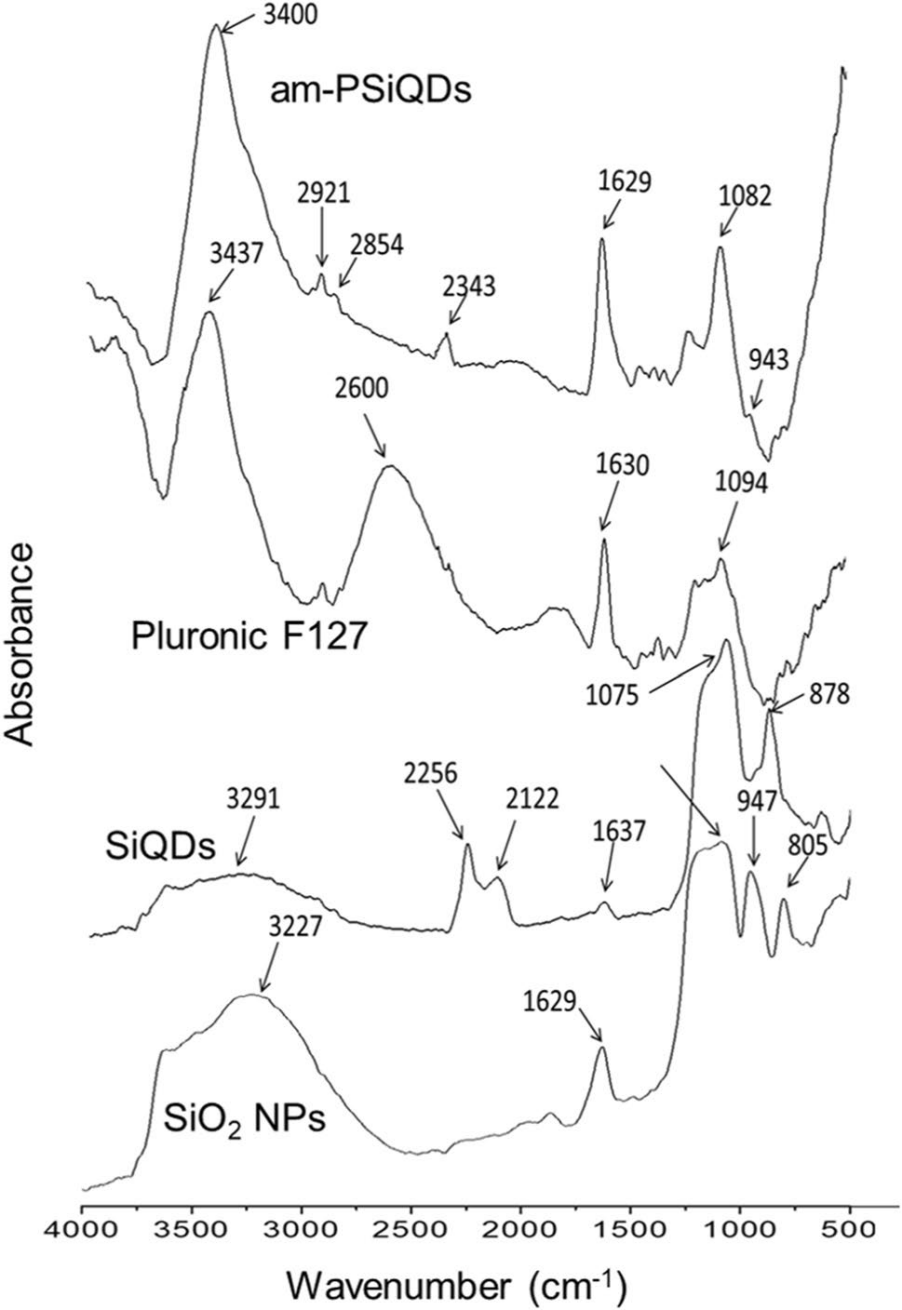
The FTIR spectra of SiO_2_ nanoparticles, SiQDs-De, Pluronic F127, and am-PSiQDs.

The FTIR analysis (Fig. 2) found that the peaks located around 878 cm^−1^ and 1075 cm^−1^, which are caused by Si–O-Si bonds, were significantly reduced when PSiQDs were used^36^. This reduction in peak intensity was compared to the peak intensity obtained with SiO_2_ NPs and SiQDs that have a native oxide layer on their surface. The peaks between 2122 and 2256 cm^−1^ were caused by the Si–H bonds. The peaks at 1630 and 3437 cm^−1^ were caused by the bending of O–H and the stretching of SiO_2_ bonds. The 1629 and 3400 cm^−1^ peaks were less intense for PSiQDs than Si-NPs. The SiQDs spectra exhibited the characteristic features of both SiQDs and F127. The peak at 1094 cm^−1^ is caused by silicon with minimal oxide, and the peak at 1629 cm^−1^ is associated with F127's C–C bond. The peaks observed at 1082 cm^−1^ and 3400 cm^−1^ in F127 suggest the successful modification of the surface of SiQDs. These peaks indicate the presence of the ether bond and the stretching of the hydroxyl group. The FTIR spectra of all three-surface modified SiQDs are shown in Figure S3.

### Synthesis and optical characterization of SiQDs with varied surface charges

In the field of biomedicine, the near-infrared region is the best option to consider due to its unique properties. This region offers a lower absorption rate and less autofluorescence from the tissues, making it an ideal choice for biomedical applications^37^. Various organic molecules in human tissues can absorb and emit light in the UV to visible range^38^. The NIR-emitting QDs are desirable for deep-tissue imaging in vivo due to improved tissue penetration of light and decreased tissue autofluorescence. The desirable range of NIR is 700–950 nm and 1200–1700 nm. Cutting-edge scientists are currently enhancing fluorescent QDs to emit and absorb light with utmost precision within the transparency window of biological tissues, thereby enabling accurate biological imaging^39^. NIR-I windows enable deeper penetration in biological tissues by reducing light scattering and absorbance. To achieve better results with NIR imaging, it is recommended to carefully choose the optical transmission window of the skin. Specifically, using wavelengths between 750 and 940 nm can lead to more accurate and reliable imaging outcomes. NIR-II windows enable tissue imaging > 2 cm depth at λ_em_ = 1000 -1700 nm.

**Figure 3:**
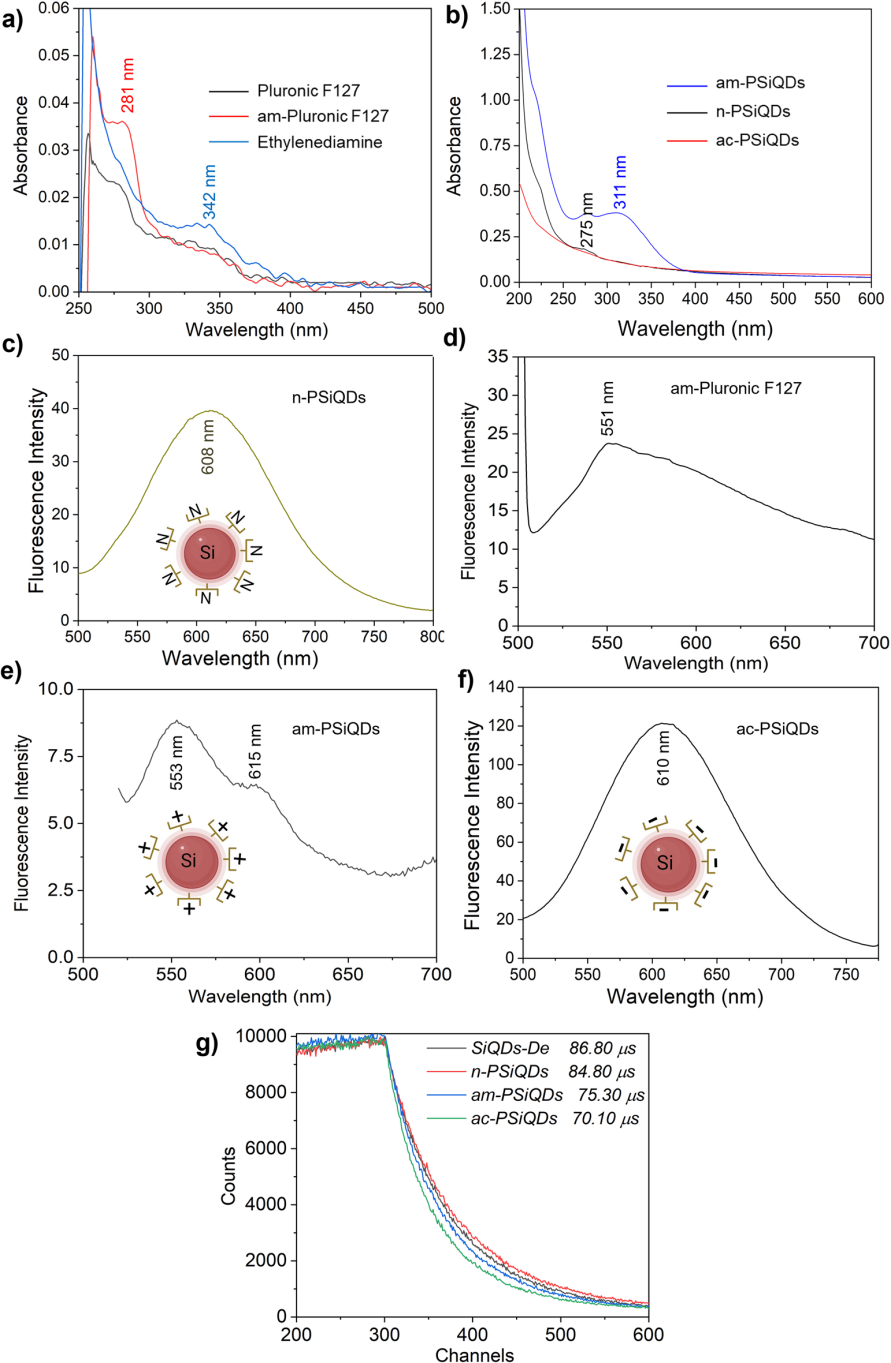
UV–visible absorption spectra of Pluronic F127, am-Pluronic f127, and Ethylenediamine (a). and am-PSiQDs, n-PSiQDs, and ac-PSiQDs (b), Fluorescence emission spectra of n-PSiQDs (c), am-Pluronic f127 (d), am-PSiQDs (e), and ac-PSiQDs (f) with 400 nm excitation. Fluorescence lifetime profiles of SiQDs-De and all three surface-modified SiQDs (g).

We prepared three different types of surface-modified PSiQDs, including amine and acrylate. In Fig. 3a, we showed absorption spectra of Pluronic F127, am-Pluronic F127, and ethylenediamine for reference. Figure 3b shows the absorption spectra of amine-capped PSiQDs (am-PSiQDs) with absorbance around 325 nm due to amine modification, acrylate-capped PSiQDs (ac-PSiQDs), and neutral surface PSiQDs (n-PSiQDs) are featureless. Figure 3c,e,f show the fluorescence emission of n-SiQDs / am-PSiQDs / ac-PSiQDs with 400 nm excitation wavelengths. The peak of fluorescence emission for n-SiQDs and ac-PSiQDs is centered at 600 nm. In the case of am-PSiQDs, the emission spectrum is broadened due to amine modification. Figure 3d shows the emission profile of ethylenediamine with a maximum peak around 560 nm. PL signals peaking at 560 nm originate from amine-pluronic. Also, the absorption as a shoulder appearing between 270 and 350 nm is possibly due to the presence of ethylenediamine. There is less difference in the magnitude and shape of PL decay curves for those samples. We evaluated the fluorescence quantum yield for am-PSiQDs, ac-PSiQDs, and n-PSiQDs: 20.8%, 19.7%, and 14%, respectively. Before modification with Pluronic F127, the SiQDs-De exhibited a quantum yield of 45%. We have carried out the measurement of fluorescence lifetime for all three PSiQDs and SiQDs-De (Fig. 3g). The fluorescence lifetime profile of surface passivated SiQDs was influenced by Pluronic F127 modification (86.80 to 70.10 µs). The three samples were excited using a pulsed LED of 370 nm wavelength. The decay times are similar to SiQD-De and smaller than hydrogen-terminated SiQD. That's why QDs are protected by decane monolayers because of the covalent linkage between surface Si atoms and terminal carbon atoms of decane. In addition, covering QD with the functionalized Pluronic molecule does not affect PL property. Although the fluorescence quantum yield is not high, it is still suitable for biological imaging and drug delivery^40–43^.

### Translational potential of the SiQDs

QDs have the potential to replace antibodies that are commonly used for staining cell surface markers. This is due to their higher stability and more affordable prices. However, in order for QDs to recognize the target markers, they need to be functionalized with specific targeting ligands. SiQDs are a safer alternative as they do not contain toxic heavy metal ions such as cadmium (Cd^2+^), mercury (Hg^2+^), and lead (Pb^2+^). SiQDs can also adjust the wavelength of light emitted from visible to NIR wavelengths. It has been reported that silicon nanocrystals break down to silicic acid upon exposure to biological fluids. The synthesized PSiQDs are highly stable when exposed to biological fluids such as PBS buffer and media. QDs have a higher surface-to-volume ratio than mesoporous silica nanoparticles, which means they have more surface area per unit of volume. Additionally, quantum dots are self-illuminating when exposed to UV light, whereas mesoporous silica requires a fluorescent probe for tracking. Finally, the particles' sizes also differ; quantum dots are less than 8 nm in size, while SiO_2_ nanoparticles are larger, with a size of more than 30 nm. We believe that these materials will prove useful for NIR bio-imaging and drug delivery in the future^47^.

### Evaluation of cellular uptake and cytotoxicity of PSiQDs with diverse surface charges

**Figure 4:**
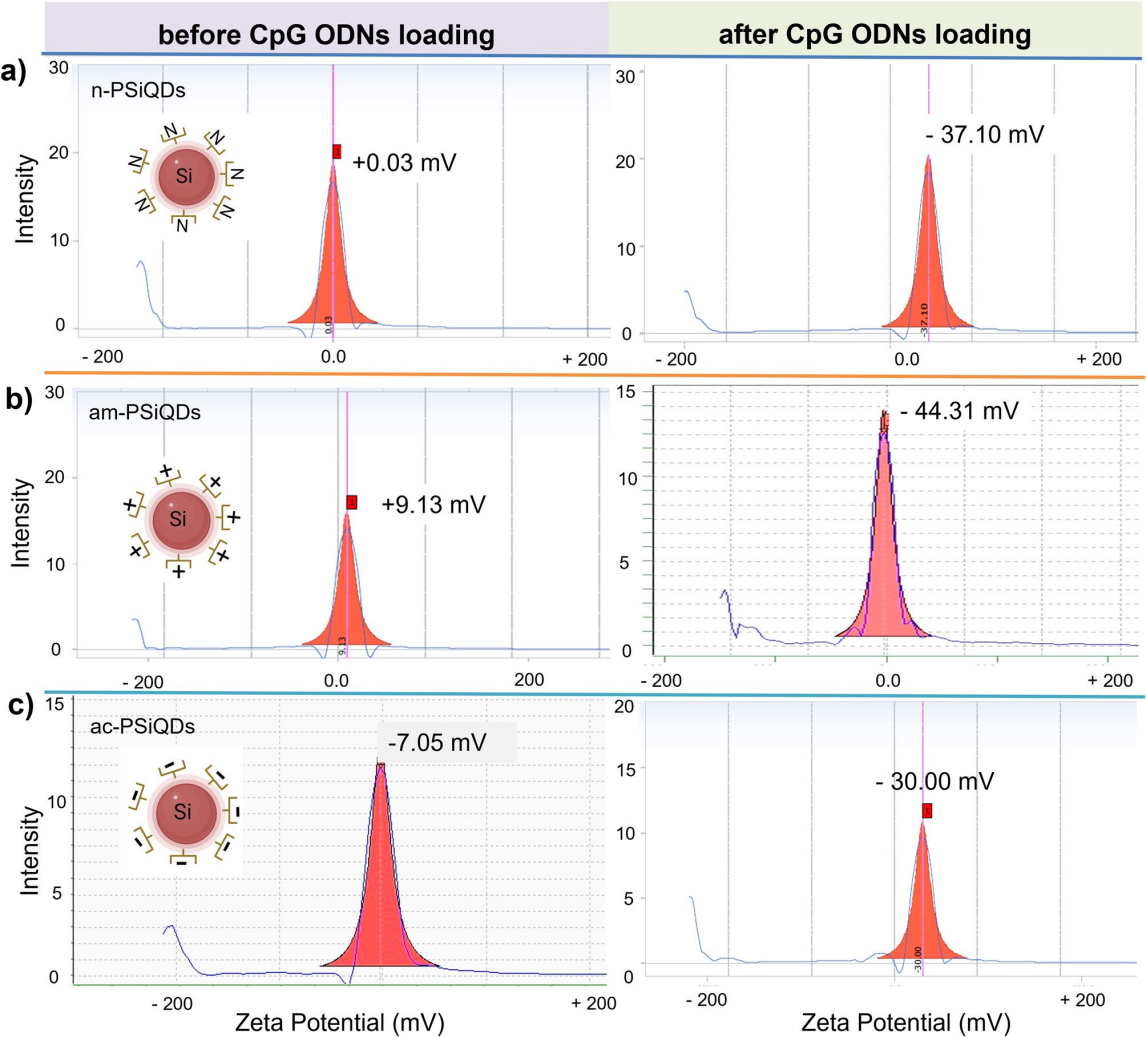
The n-PSiQDs, am-PSiQDs, and ac-PSiQDs surface charges before and after CpG ODNs loading.

**Figure 5:**
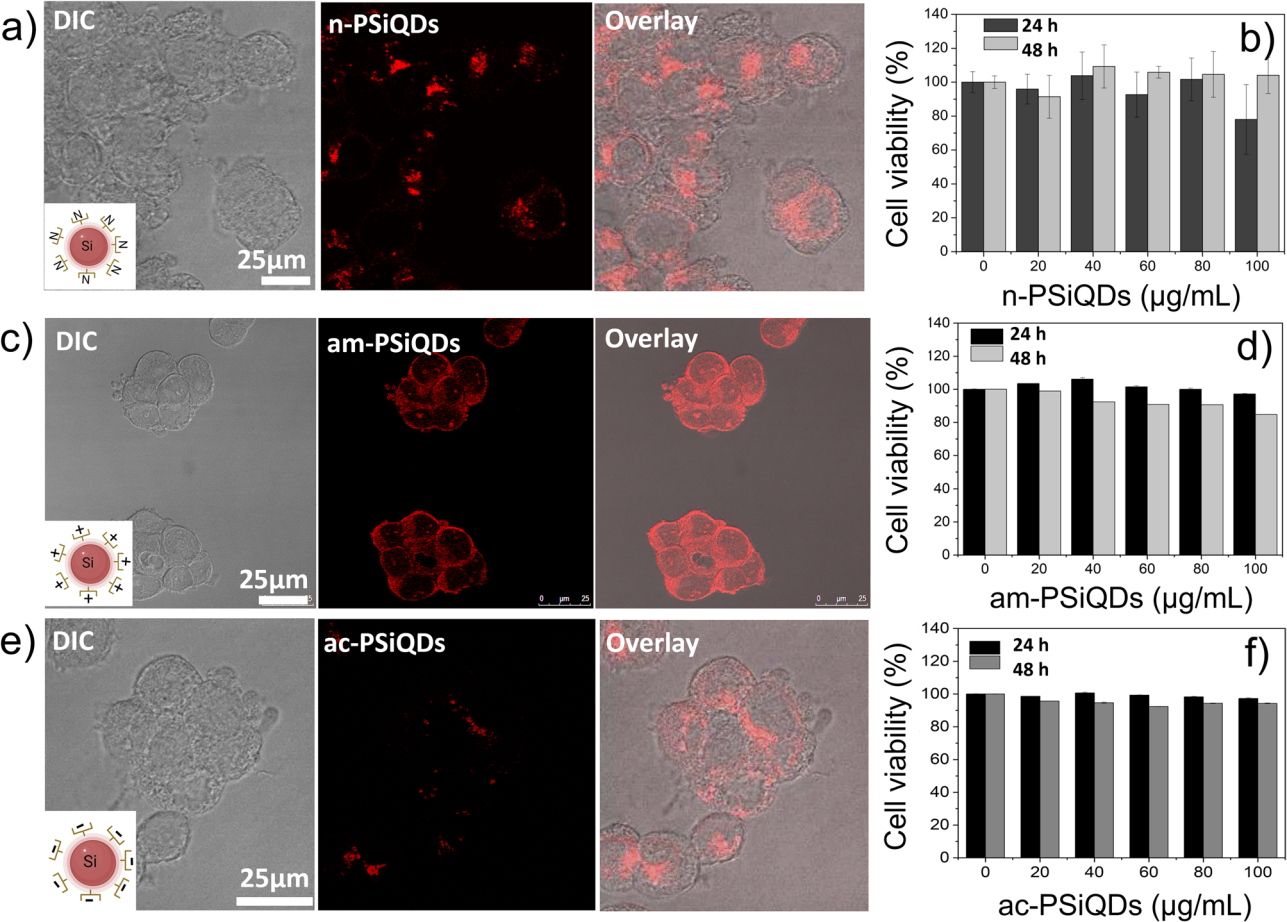
PBMC cellular uptake (a, c, e) and cytotoxicity (b, d, f) of surface-modified SiQDs.

Pluronics are copolymers comprised of two hydrophilic PEO chains linked via a central hydrophobic PPO. Pluronic-based nanomedicine has proven to be an effective method for delivering cancer drugs^44,45^. The surface charge measurements reveal that am-PSiQDs possess a charge of + 9.13 mV (Fig. 4b), while ac-PSiQDs exhibit a charge of − 7.05 mV (Fig. 4c), and those with a n-PSiQDs display a value of approximately + 0.03 mV (Fig. 4a). These variations in charge indicate that the surface charge of SiQDs is significantly influenced by the type of Pluronic F127 used in their modification. Next, we conjugated CpG ODNs to the surface of PSiQDs. After incubating PBMC cells with prepared PSiQDs for 24 h, we observed efficient uptake by the cells without any changes in cell morphology. (Fig. 5a,b,c). The overlay images clearly indicated the localization of the materials around the cell nucleus. In addition, the biocompatibility of the prepared SiQDs was tested by assessing cell viability in PBMC cells. After incubation with varying concentrations (0–100 µg/mL) of SiQDs for 24 & 48 h, no significant cytotoxicity was detected (Fig. 5b,d,f).

### CpG ODNs loading to pluronic modified SiQDs.

**Figure 6:**
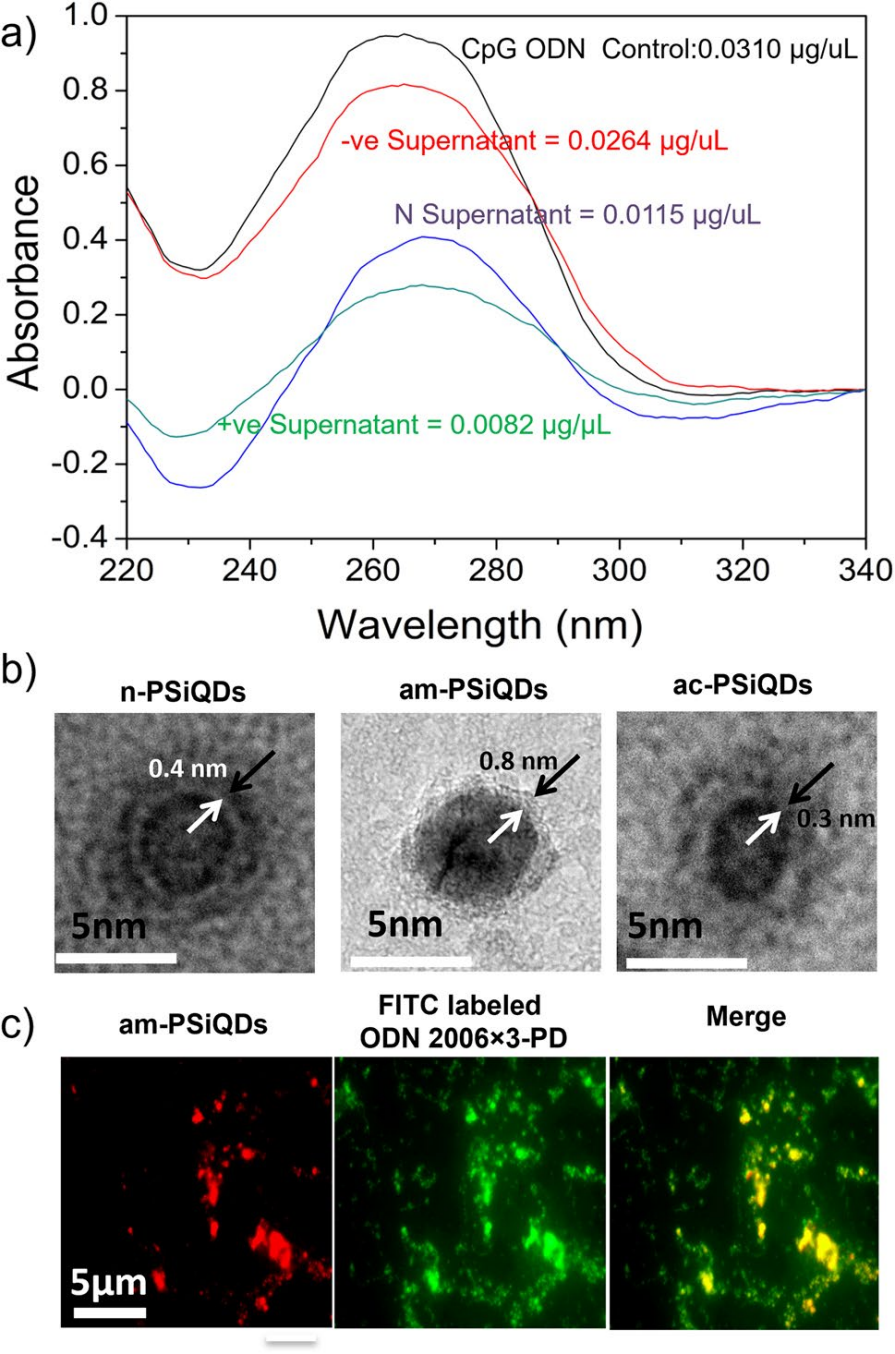
UV–Visible spectra show the supernatant concentrations of the CpG ODNs (a). TEM image of surface-modified SiQDs loaded with CpG ODNs. Arrows indicate the CpG ODNs layer (b). Confirmation of FITC labeled CpG ODNs on the surface of the am-SiQDs (c).

The uptake of CpG ODNs by PSiQDs was quantified using a nanodrop spectrometer, allowing for the determination of CpG ODN concentrations in the supernatant (Fig. 6a). TEM images revealed that the CpG ODNs formed a coating on the PSiQDs surface with a thickness ranging 0.3 to 0.8 nm according to the surface charge (Fig. 6b). Following the binding of CpG ODNs, the surface charge of the PSiQDs exhibited significant changes from + 0.03 mV to − 37.32 mV for the n-PSiQDs, from + 9.13 mV to − 44.31 mV for the am-PSiQDs, and − 7.05 mV to − 30.00 mV for ac-PSiQDs (Fig. 4). The uptake of CpG ODNs by PSiQDs varied according to the PSiQDs surface charge: 4.6 µg/mg (QDNs/QDs) for ac-PSiQDs, 22.8 µg/mg positive SiQDs and 19.5 µg/mg for nSiQDs. The amount of CpG ODNs bound to am-PSiQDs and n-PSiQDs with different surface charges was found to be not significantly different. The results of the study suggest that the use of am-PSiQDs can effectively achieve the maximum uptake of CpG ODNs by electrostatic attraction. In the case of ac-PSiQDs, the uptake efficiency is lower than am-PSiQDs and n-PSiQDs due to the opposite charge. In this regard, we used FITC-labeled ODNs on the surface of PSiQDs and confirmed by inverted fluorescence microscopy. The overlay image confirms the presence of the CpG ODNs on the surface of the PSiQDs (Fig. 6c).

### Cytokine production by CpG ODNs bound onto PSiQDs.

**Figure 7:**
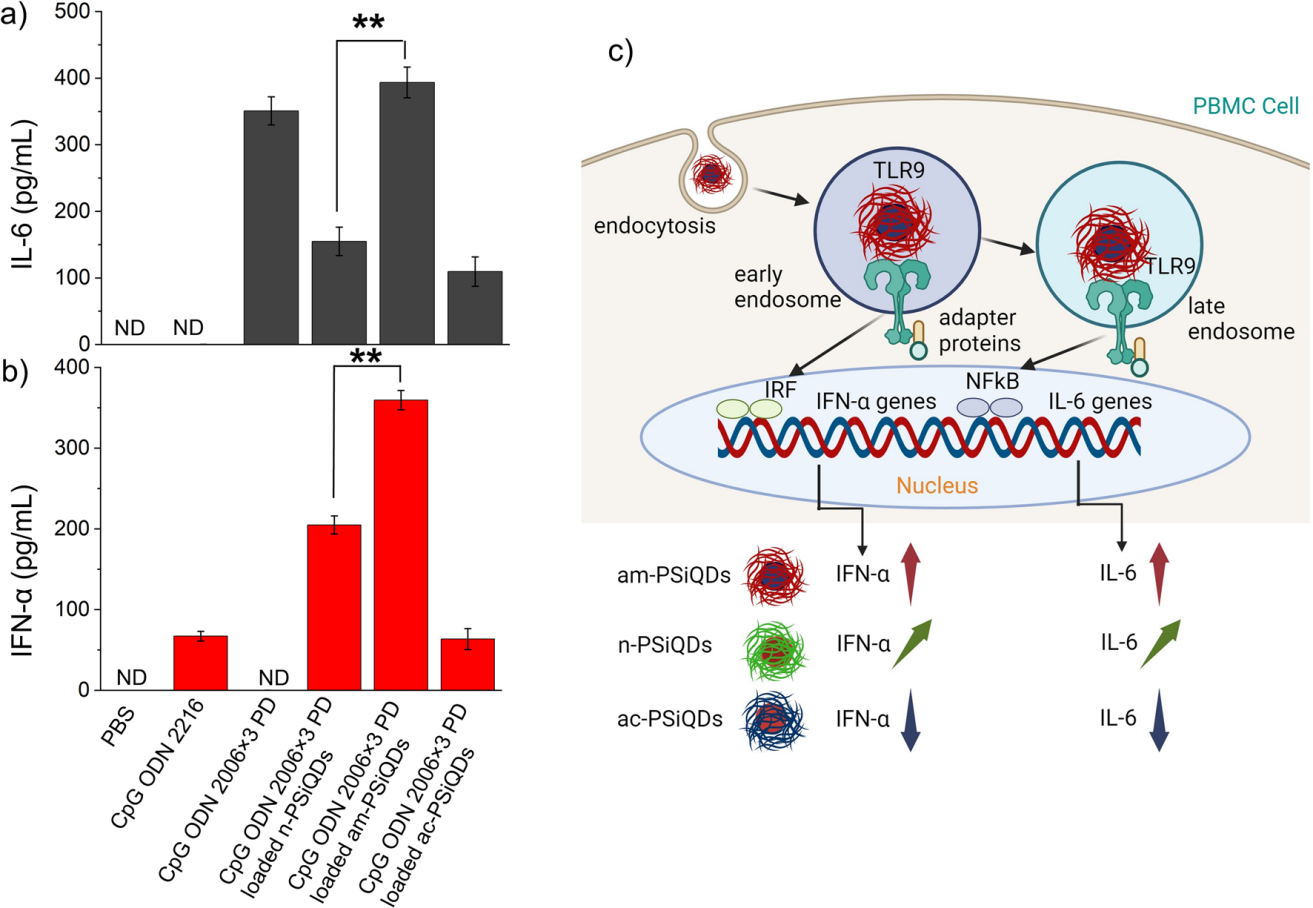
The levels of cytokines induced by CpG ODNs bound onto SiQDs (a, b). ND, not detected, n = 3, **, *p* < 0.05. The schematic diagram shows levels of cytokines induced by CpG ODNs bound onto am-PSiQDs, n-PSiQDs, and ac-PSiQDs (c).

Our previous report has conclusively shown that the activation of TLR9 is entirely dependent on the type and length of ODNs, which are, without a doubt, short DNA sequences^11,12,46^. The study showed that different ODN sequences and sugar types can lead to varying amounts of the cytokine IL-6. We also discovered that free CpG ODNs, which are similar to class B CpG ODNs, can activate TLR9 and induce the production of IL-6 through the activation of NFkB. However, this activation did not lead to the production of IFN-α. When positively charged SiQDs were used to electrostatically bind CpG ODNs, the observed IL-6 and IFN-α production levels were the highest. The results showed that when the surface of SiQDs was modified with negative or neutral particles, there was a significant reduction of IL-6 induction. This suggests that surface modification of SiQDs can potentially be used as a strategy to regulate the immune response and minimize inflammation (Fig. 7a,b). Positively charged SiQDs (am-PSiQDs) increased IFN-α induction due to high CpG ODN uptake, and neutral surfaced SiQDs (n-PSiQDs) also induced IFN-α significantly due to multimerization of CpG ODNs on the PSiQDs. The schematic diagram (Fig. 7c) shows the cytokine production levels depending on the surface-modified PSiQDs. The upward aero indicates maximum level production, and the downwards aero represents lower cytokine production. Medium-level production shows diagonal aero.

### Cellular localization of PSiQDs loaded with CpG ODNs

**Figure 8:**
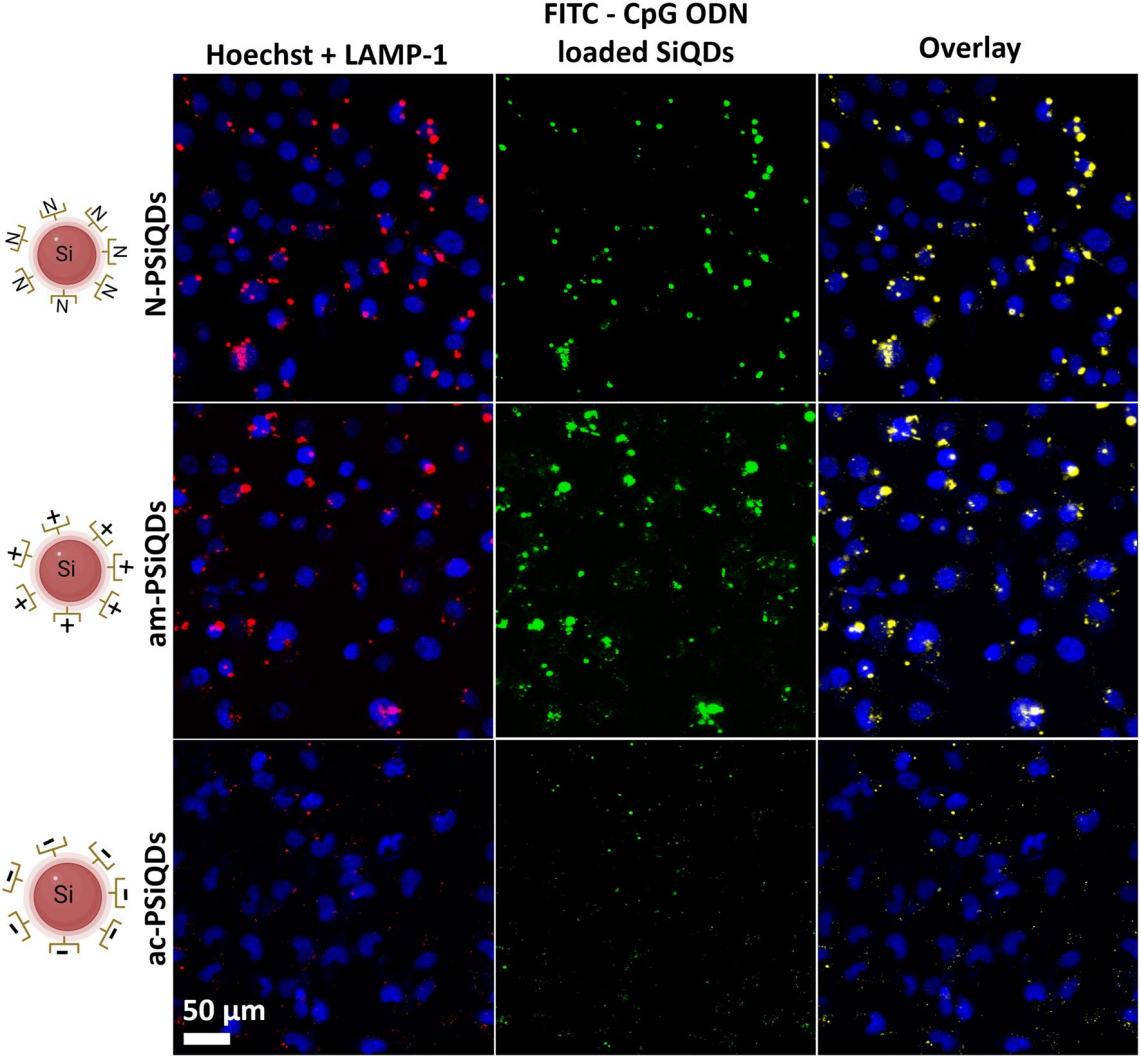
PBMC cellular uptake of surface-modified SiQDs loaded with CpG ODNs. The blue color indicates the cell nucleus, while the green color indicates CpG ODNs labeled with FITC, and the yellow color represents the overlay of LAMP-1 and FITC-labeled CpG ODNs.

We investigated the intracellular positioning of CpG ODNs on SiQDs that have been modified with Pluronic. PSiQDs loaded with CpG ODNs were found to be localized within late endosomes that expressed LAMP-1 (Fig. 8). The localization of CpG ODNs varied depending on the surface charge of the SiQDs. When comparing positive, neutral, and negative surface-modified SiQDs, confocal fluorescence images showed that high-density light was emitted from the positive surface-modified Si-QDs (am-PSiQDs) with FITC-labeled CpG ODNs. The IL-6 and IFN-α inductions are directly related to the amount of ODN uptake by the endosome in the PBMC cells.

## Conclusion

This article outlines the preparation of three distinct surface-charged SiQDs-F127 (+ 0.03, + 9.13, − 7.05 mV), varying from negative to positive charges. Depending on the surface charges, the amount of CpG ODNs also varies; positively charged materials take up more ODNs (− 44.31 mV) than neutral (− 37.10 mV) and negatively charged (− 30.00 mV) materials due to electrostatic attraction. We assessed the viability of the cells, as well as their uptake and localization in endosomes. PSiQDs with a positive charge are more localized and induce higher levels of cytokines, including IL-6 and IFN-α, due to increased endosomal localization compared to other surface-charged PSiQDs. The results indicate that the surface charges of the nanoparticle play a significant role in delivering CpG ODN2006 × 3-PD and inducing cytokines.

### Supplementary Information

Supplementary Information.

## Data Availability

The data supporting this study's findings are available from the corresponding author (S.C) upon reasonable request.
