# Supplementary material for: Surface charge-dependent cytokine production using near-infrared emitting silicon quantum dots
Source: Sci Rep. 2024 Apr 26;14:9618. doi: 10.1038/s41598-024-60536-2 (PMC11053057; doi:10.1038/s41598-024-60536-2)
Supplement: Supplementary file 1 — Supplementary Information. [file 41598_2024_60536_MOESM1_ESM.docx]

**Supporting Information**

**Surface charge-dependent cytokine production using**

**near-infrared emitting silicon quantum dots**

Shanmugavel Chinnathambi^a*^, Naoto Shirahata ^b,c,*^, Pooria Lesani ^d,e^, Vaijayanthi Thangavel^a,^ and Ganesh N. Pandian ^a*^

*^a^Institute for Integrated Cell-Material Sciences, Institute for Advanced Study, Kyoto University, Kyoto, 616-8510 Japan*

*^b^Graduate School of Chemical Sciences and Engineering, Hokkaido University, Kita 13, Nishi 8, Kita-ku, Sapporo 060-0814, Japan*

*^c^International Center for Materials Nanoarchitectonics, National Institute for Materials Science, Namiki, Tsukuba 305-0044, Japan.*

*^d^School of Science, STEM College, RMIT University, Melbourne, VIC 3000, Australia*

*^e^Koch Institute for Integrative Cancer Research, Massachusetts Institute of Technology, Cambridge, MA 02139, USA*

Corresponding email address: [chinnathambi.shanmugavel.8s@kyoto-u.ac.jp](mailto:chinnathambi.shanmugavel.8s@kyoto-u.ac.jp) ; [SHIRAHATA.Naoto@nims.go.jp](mailto:SHIRAHATA.Naoto@nims.go.jp); namasivayam.ganeshpandian.5z@kyoto-u.ac.jp


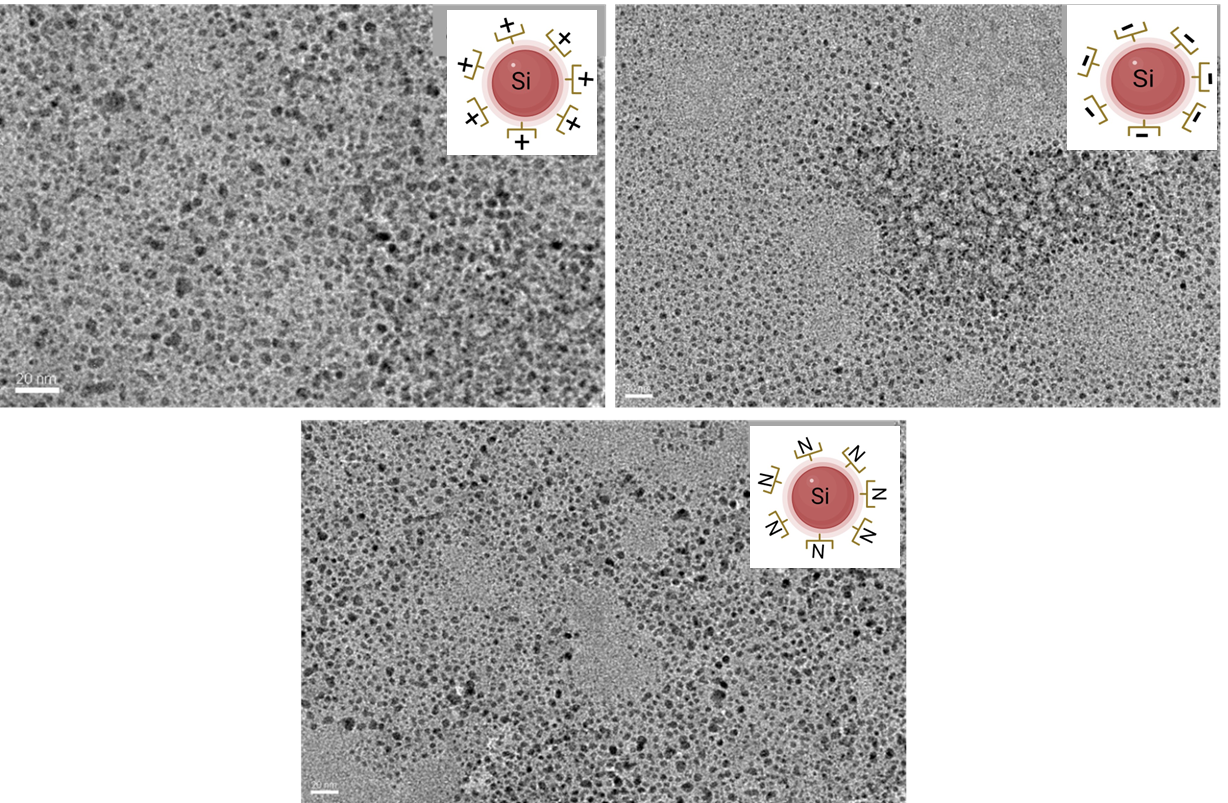


**Figure. S1** The HR-TEM images of am-PSiQDs, ac-PSiQDs and n-PSiQDs. The average size of the Silicon Quantum Dots (SiQDs) ranges from 2 to 6 nm.


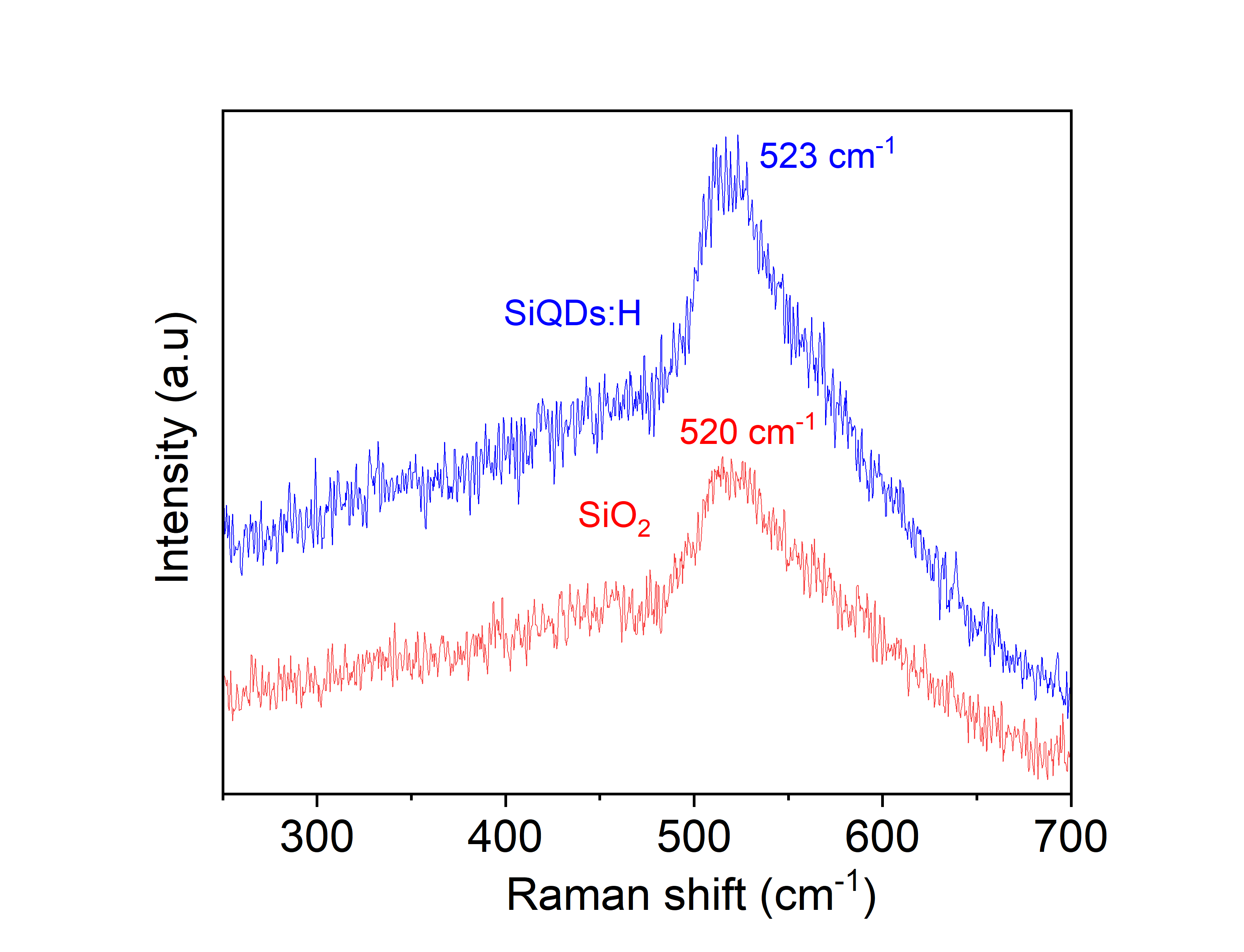


**Figure. S2** The Raman spectra of hydrogen-terminated SiQDs.


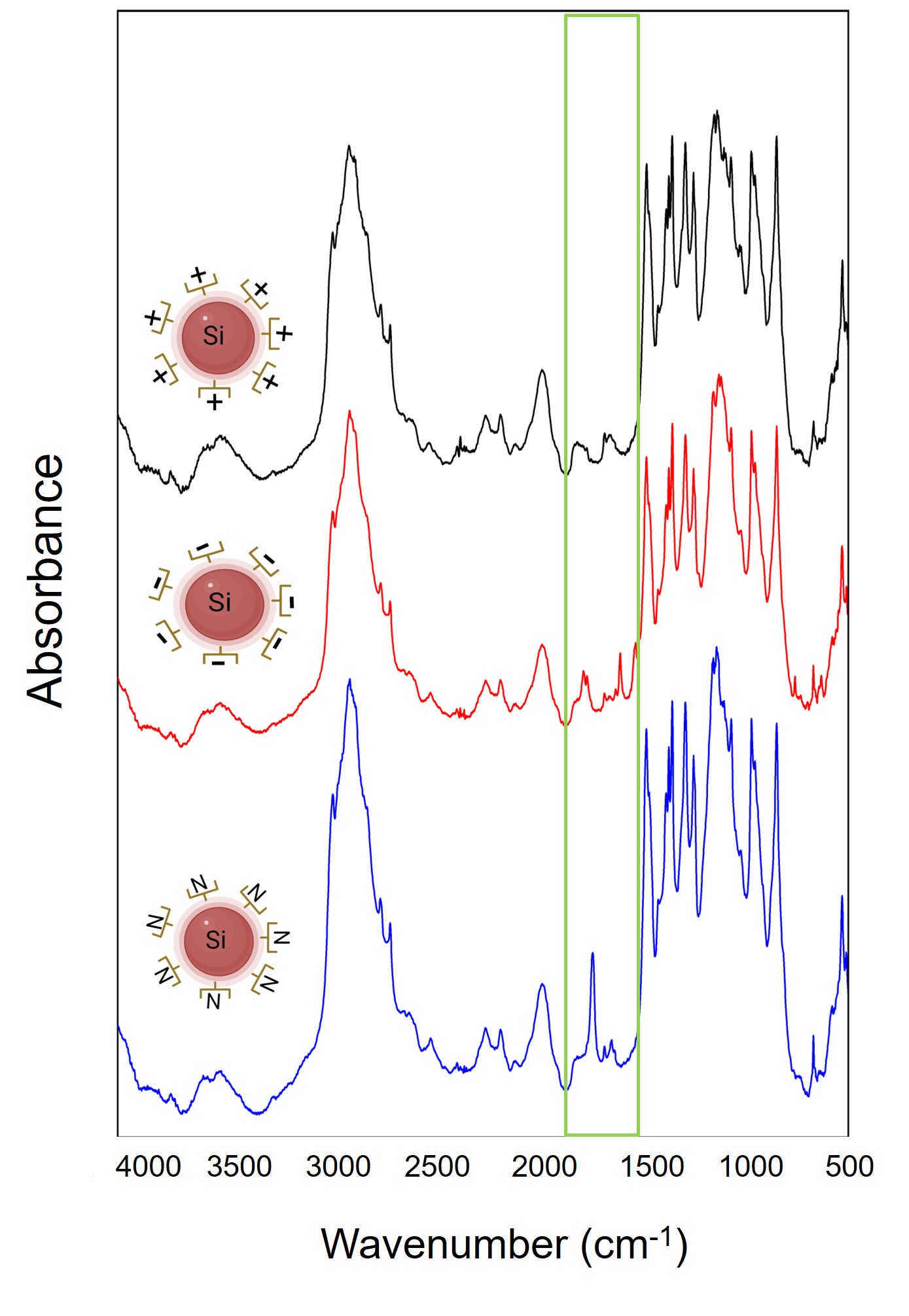


**Figure. S3** The FTIR spectra of am-PSiQDs, ac-PSiQDs and n-PSiQDs.
